# Does bilingual experience affect early visual perceptual development?

**DOI:** 10.3389/fpsyg.2014.01429

**Published:** 2014-12-11

**Authors:** Christina Schonberg, Catherine M. Sandhofer, Tawny Tsang, Scott P. Johnson

**Affiliations:** Department of Psychology, University of CaliforniaLos Angeles, CA, USA

**Keywords:** bilingualism, language, cognition, perception, infancy, development

## Abstract

Visual attention and perception develop rapidly during the first few months after birth, and these behaviors are critical components in the development of language and cognitive abilities. Here we ask how early bilingual experiences might lead to differences in visual attention and perception. Experiments 1–3 investigated the looking behavior of monolingual and bilingual infants when presented with social (Experiment 1), mixed (Experiment 2), or non-social (Experiment 3) stimuli. In each of these experiments, infants' dwell times (DT) and number of fixations to areas of interest (AOIs) were analyzed, giving a sense of *where* the infants looked. To examine *how* the infants looked at the stimuli in a more global sense, Experiment 4 combined and analyzed the saccade data collected in Experiments 1–3. There were no significant differences between monolingual and bilingual infants' DTs, AOI fixations, or saccade characteristics (specifically, frequency, and amplitude) in any of the experiments. These results suggest that monolingual and bilingual infants process their visual environments similarly, supporting the idea that the substantial cognitive differences between monolinguals and bilinguals in early childhood are more related to active vocabulary production than perception of the environment.

## Introduction

Visual attention and perception develop rapidly during the first few months after birth, and these behaviors are critical components in the development of language and cognitive abilities. Visual attention, habituation, degree of novelty preference, and visual recognition in infancy have been found to predict cognitive development, language development, and IQ in early childhood (Rose et al., 1989, 1991; McCall and Carriger, 1993; Rose and Feldman, 1995; Colombo et al., 2004). Furthermore, receptive language and expressive language both partially mediate the relation between looking behavior in infancy and IQ in childhood (Rose et al., 1991). This paper examines how early language experience may influence looking behavior in monolingual and bilingual infants prior to the development of extensive receptive and expressive vocabulary and discusses the implications thereof.

Previous research has shown that language experience—in particular, the type and number of languages one speaks—has broad effects on performance on a variety of tasks in childhood and adulthood. There is evidence to suggest that fluency in multiple languages (i.e., bi- or multilingualism) leads to many cognitive consequences, including advantages in executive function (e.g., Bialystok and Martin, 2004; Carlson and Meltzoff, 2008; Gold et al., 2013), working memory (Morales et al., 2012), perspective-taking (Greenberg et al., 2013), and symbolic flexibility (Thom and Sandhofer, 2014). Cognitive effects of bilingualism are found throughout the lifespan—bilingual 18-month-olds outperform their monolingual peers in memory generalization tasks (Brito and Barr, 2012), and in older adults, bilingual experience can serve to delay the onset of dementia symptoms (Bialystok et al., 2007). However, the robustness of these effects is still under investigation, as cognitive advantages for bilinguals are not consistently found (e.g., Antón et al., 2014; Duñabeitia et al., 2014). Regardless of whether one is monolingual or bilingual, native language fluency leads to language-based differences in cognition and perception. Memory, categorization, and visual and auditory perception are just a few examples of the domains that are influenced by one's native language (e.g., Werker and Tees, 1984; Davidoff, 2001; Boroditsky, 2003; Davidoff et al., 2008).

The effects of native language on attention and perception (e.g., color or phoneme perception) are most likely direct results of frequent, extended use of a symbolic system that frames the environment in a particular manner. That is, monolinguals use one symbolic system to communicate and frame their environments, and bilinguals use two systems. However, even brief exposure to linguistic materials can influence perceptual and conceptual decisions (Goldstone, 1994; Dils and Boroditsky, 2010). Importantly, language influences visual attention and perception both when language is produced as well as when language is processed.

Even before infants begin to produce words, their looking behaviors are influenced by individual experiences in processing language. As infants develop and become more attuned to their native language(s), they become less able to discriminate between visual concepts that are not labeled in the language(s) with which they have the most experience (Choi et al., 1999; Göksun et al., 2011). Infants who are raised in dual-language environments, regardless of their specific languages, are better able to visually discriminate spoken languages (i.e., silent videos of people speaking different languages) than their monolingual peers (Weikum et al., 2007). This effect is found even when the bilingual infants have no experience with the languages being shown (Sebastián-Gallés et al., 2012). Thus, language experience influences infants' looking behavior on language-related tasks.

In addition to influencing language-related tasks, language experience also plays a role in older children's distribution of visual attention during non-linguistic tasks. For example, bilingual children, as compared to their monolingual peers, allocate more attention to an interaction partner's referential gestures (Yow and Markman, 2011). If learning multiple language systems affects older children's looking behavior in non-linguistic tasks, then exposure to multiple language systems may affect visual attention and perception during these tasks in infancy as well. Therefore, we expect that infants from bilingual backgrounds may show different patterns of looking behavior than infants from monolingual backgrounds.

One reason we might expect to find different patterns of looking behavior for bilingual infants is that they receive a greater variety of input due to the nature of their linguistic environments. Monolingual infants have been shown to be influenced by variable input in that they attend to objects differently depending on how the objects are labeled (Xu, 2002; Xu et al., 2005). Infants growing up in bilingual environments are exposed not only to different labels, but to two entirely different language systems. In much the same way that diverse labels affect how monolinguals attend to objects, the diversity of input that bilingual infants receive could lead to changes in their attentional patterns. We reasoned, therefore, that infants from bilingual environments may exhibit a broader or more general attentional style than infants from monolingual environments, possibly reflecting a precursor to the bilingual advantage in monitoring found later in development (e.g., Costa et al., 2008). That is, due to bilingual infants' greater variety of input, they may attend to the environment more generally. Looking patterns that may indicate monitoring or broader attention include dividing looking time between different stimuli and making fixations to a greater number of unique areas of interest (AOIs).

To date, a great deal of the research on bilingual input in infancy has focused on tasks and outcomes that are specifically language-related, such as phoneme perception (e.g., Byers-Heinlein and Fennell, 2014), visual speech discrimination (e.g., Weikum et al., 2007), and language acquisition (e.g., Werker and Byers-Heinlein, 2008). However, we are aware of no published work addressing the implications of bilingual input in infancy beyond language learning. This paper examines the extent to which language experience influences looking behavior in both linguistic and non-linguistic tasks and asks whether monolingual and bilingual infants are the same or different in how they process their visual environments.

In the following four experiments, we address research questions about the effect of language experience on looking behavior in monolingual and bilingual infants by analyzing existing data sets (Nguyen et al., 2012; Gluckman and Johnson, 2013; Kim and Johnson, 2013, 2014). In this preverbal context, we define infants as bilingual if they are consistently spoken to in two languages (also called a “crib bilingual”). Experiment 1 examines looking behavior to social stimuli (two side-by-side faces), Experiment 2 examines looking behavior to social and non-social (mixed) stimuli, and Experiment 3 examines looking behavior to non-social stimuli. We focus on the social/non-social aspects of the stimuli because we hypothesized that infants from bilingual environments may pay special attention to social partners. Compared to monolingual infants, bilingual infants may have more variety in their social interactions and a greater need to attend to a partner's characteristics (e.g., culture, language) in order to select appropriate expectations (and, at later ages, behavior) for the interaction. In all three experiments, we collected dwell time, fixation, and saccade data using eye-tracking methods.

Experiment 4 investigates global looking behavior across all stimuli types by pooling together the saccade data collected in Experiments 1–3. This measure will answer the question of whether monolingual and bilingual infants engage in different patterns of eye movements when processing visual information. For example, bilingual infants may make saccades between several objects in rapid succession, quickly scanning the environment, while monolingual infants may make fewer saccades, focusing on only a small number of objects or screen locations.

## Experiment 1A

Because we were interested in whether monolingual and bilingual infants show different patterns of visual attention, we examined the possibility that language background played a role in the Kim and Johnson (2014) study of infants' looking to infant-directed (ID) and adult-directed (AD) faces in the presence of ID or AD speech. In the same way that ID speech is overly exaggerated and has a wider prosodic range compared to AD speech, adults' ID facial expressions are larger and more animated in comparison to their AD facial expressions. In this experiment, infants saw side-by-side videos of ID and AD faces and heard asynchronous ID speech, asynchronous AD speech, or silence.

Based on our hypothesis that crib bilinguals may have a special tendency to divide their attention between social partners, we predicted that bilingual infants, relative to monolingual infants, would tend to look more equally at both faces when no speech was played. Monolingual infants, on the other hand, might spend the majority of the time looking at one face during the no speech condition. In the conditions in which ID or AD speech were played, we hypothesized that bilingual infants would be more likely than monolinguals to look at the face that matched the audio (i.e., AD face in the presence of AD speech; ID face in the presence of ID speech). We reasoned that bilingual infants would be better than their monolingual counterparts at this task because they have more experience matching different types of language to different people. However, if ID faces convey more information or are more interesting than AD faces, infants from both language backgrounds may prefer to look at the ID face regardless of speech condition. To assess infants' looking behavior, we measured their dwell time and number of fixations to each face.

### Method

#### Participants

Participants were recruited from a university participant database. Eight monolingual (3 girls) and 13 bilingual (4 girls) 3-month-old infants (*M* = 3.1 months, *SD* = 0.25 months) participated in this experiment. Fifteen infants were excluded from the original data set due to lack of language background information.

***Language background***. Infants were classified as having either monolingual or bilingual language experience based on the languages listed in their database record. To verify the accuracy of the database, a subset of parents was contacted via email and asked to fill out a language background questionnaire (Appendix A). The questionnaire asked parents to specify, for each person with whom the infant regularly interacted, the following information: the languages used with the infant, fluency levels in those languages, and the amount of time spent with the infant per week.

To determine whether an infant came from a monolingual or bilingual background, we used two criteria. For parents who answered our questionnaire, we used the percentages given in question three to make classifications. Infants with ≤ 10% exposure to a second language (L2) were classified as monolingual; infants with >10% L2 exposure were classified as bilingual. Monolingual infants had an average of 1.5% L2 exposure (range: 0–5%), and bilingual infants had an average of 50% L2 exposure. For parents who could not be contacted or did not return the questionnaire, we classified infants as monolingual or bilingual based on how many languages were listed in their database record, because we found such information in the database to be consistent with the parent questionnaire. We compared the questionnaire responses to the languages listed in the database for those participants and found 94% agreement between the database and survey responses.

#### Design

This experiment used a 2 × 2 × 3 mixed design. Language group (monolingual or bilingual) served as the between-subjects factor, and face type (ID or AD) and speech type (ID, AD, or silence) were the within-subjects factors. Dependent variables of interest were dwell time (DT) and number of fixations to each face.

#### Stimuli and apparatus

A Tobii 1750 eye tracker was used to record eye movements at 60 Hz with an approximate spatial accuracy of 0.5–1°. Each infant sat on a parent's lap approximately 60 cm from a 17” computer monitor with a screen resolution of 640 × 480. To calibrate each infant's point of gaze, a dynamic attention-getter was briefly shown at five points around the screen (each of the corners and the center). Calibration continued until data showing good-quality calibration, as determined by the Tobii eye tracker, for each point were collected. Before each trial, a visual attention-getter appeared in the center of the monitor to re-orient the infant's gaze to the middle of the screen.

**Figure 1:**
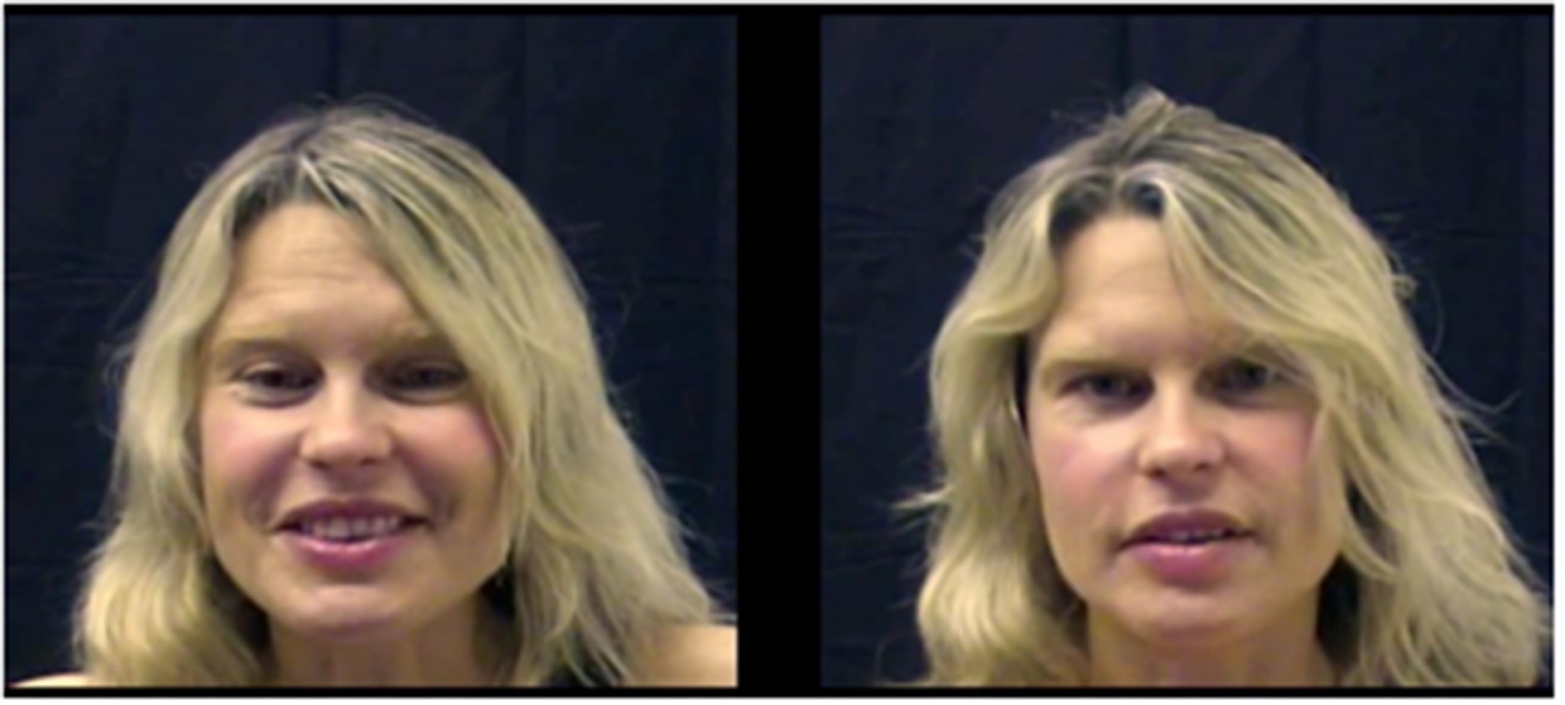
**Screen shot of the video stimuli used in Experiment 1A**. In this experiment, infants viewed both videos simultaneously on the same screen, and the videos were either played with asynchronous audio (infant-directed speech or adult-directed speech) or no audio (silence condition). Here, the infant-directed (ID) face is on the left, and the adult-directed (AD) face is on the right.

To create the stimuli, experimenters recorded videos of the same woman talking to either an adult (her husband) or an infant (her 18-month-old). The audio and video from these recordings were separated, and each video was divided into several 20-s segments. The segments were then rated as being infant-directed or adult-directed by undergraduates. The six ID and six AD video segments that had been rated as most infant-directed and most adult-directed, respectively, were paired to create six unique video pairs (see Figure 1). Audio was divided and rated as being ID or AD in a similar manner. Details of stimuli creation and data collection can be found in Kim and Johnson (2014).

#### Procedure

Infants were seated on a parent's lap for the duration of the experiment. In this experiment, infants saw side-by-side videos of the same person engaging in infant-directed or adult-directed speech. During each video trial, infants heard either a recording of infant-directed speech, a recording of adult-directed speech, or silence. Before each new trial, a visual attention-getter appeared in the center of the monitor to re-orient the infant's gaze to the middle of the screen. There were four video trials matched with each sound type, making 12 trials in all.

### Results and discussion

This experiment asked whether 3-month-old infants' documented preference for ID speech extends to ID faces and investigated how speech influences looking behavior. To control for individual differences in looking time, infants' dwell times to each face type (ID or AD) were converted to proportions (out of the total trial length). To control for individual differences in dwell time, total dwell time was used as a covariate. However, due to the fact that this variable is a time-varying covariate, the 2 × 2 × 3 omnibus ANCOVA that would normally be used for this design could not be carried out. Rather, we used a general linear mixed model, which takes into account the repeated nature of the data, to examine whether crib bilinguals would be better able to match speech and facial characteristics. The model was generated using the Linear Mixed Models procedure in SPSS. The dependent variable was proportion of dwell time, and the covariate was total dwell time (ms) spent watching the videos. The fixed effects factors in the model were language background, face type (ID or AD), and speech type (ID, AD, or silence). We included the intercept as the random effect in the model, which is a measure of variance in proportion of dwell time between participants as explained by the covariate. We used a full factorial model and were particularly interested in potential interactions between the fixed factors.

There was no significant three-way interaction for language background, face type and speech type [*F*_(2, 126)_ = 0.62, *p* = 0.541], nor were there any significant two-way interactions for face and speech type [*F*_(2, 126)_ = 0.35, *p* = 0.704], language background and speech type [*F*_(2, 126)_ = 0.214, *p* = 0.807], or language background and face type [*F*_(1, 126)_ = 0.69, *p* = 0.407]. There was not a main effect by speech type [*F*_(2, 126)_ = 0.47, *p* = 0.629], or language background [*F*_(1, 126)_ < 0.0001, *p* > 0.999]. However, we did observe a main effect of face type on proportion of dwell time on the areas of interest [*F*_(1, 126)_ = 18.91, *p* < 0.0001], in which infants, regardless of language group or speech type, looked proportionally more to the ID face (*M* = 0.093, *SD* = 0.03) than to the AD face (*M* = 0.073, *SD* = 0.04). Individual condition means are reported in Table 1. These results suggest that bilingual 3-month-olds do not differ from their monolingual counterparts in matching ID or AD voices to ID or AD faces, respectively.

**Table 1:** **Mean proportion dwell time to ID and AD faces in Experiment 1A**.

**Audio condition**		**ID face**	**AD face**
Silence	Monolingual	0.081 (0.04)	0.063 (0.04)
	Bilingual	0.095 (0.04)	0.078 (0.04)
ID speech	Monolingual	0.087 (0.03)	0.072 (0.04)
	Bilingual	0.096 (0.04)	0.078 (0.03)
AD speech	Monolingual	0.085 (0.03)	0.072 (0.02)
	Bilingual	0.107 (0.04)	0.072 (0.04)

*Standard deviations appear in parentheses*.

To examine whether monolingual and bilingual infants showed different patterns of looking to AD and ID faces, we also analyzed the mean number of fixations they made to each face type. One-way ANOVAs showed that there was no main effect of language background on mean number of fixations made to AD faces, [*F*_(1, 19)_ = 2.64, *p* = 0.121], or to ID faces, *F*_(1, 19)_ = 0.52, *p* = 0.482 (see Table 2). Thus, across the different speech conditions, monolingual and bilingual 3-month-olds did not appear to differ in how they look to ID and AD faces.

**Table 2:** **Mean number of fixations per trial to ID and AD faces in Experiment 1A**.

**Audio condition**		**ID face**	**AD face**
Silence	Monolingual	4.57 (2.64)	2.50 (0.84)
	Bilingual	4.78 (1.74)	3.98 (1.60)
ID Speech	Monolingual	4.09 (2.21)	3.31 (1.17)
	Bilingual	4.60 (1.39)	3.99 (1.39)
AD Speech	Monolingual	12.91 (6.08)	9.18 (1.69)
	Bilingual	5.18 (1.66)	3.78 (1.95)
Overall mean fixations	Monolingual	12.91 (6.08)	9.18 (1.69)
	Bilingual	14.51 (3.65)	11.67 (4.17)

*Standard deviations appear in parentheses*.

**Figure 2:**
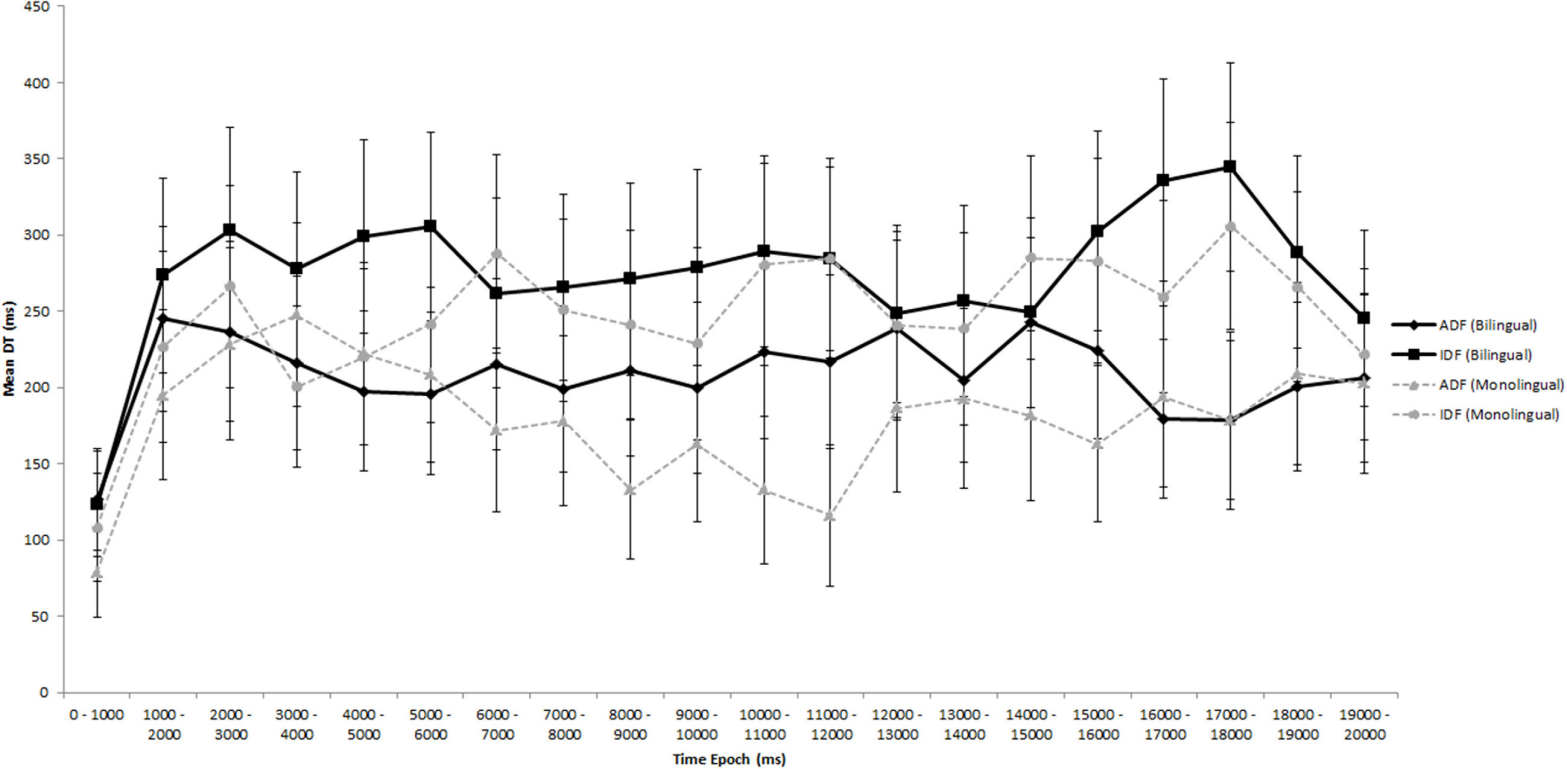
**Infants' looking patterns in Experiment 1A**. The solid black line represents bilingual infants' looking patterns to the adult-directed face (ADF) and the infant-directed face (IDF); the dotted gray line represents monolingual infants' looking patterns. Error bars represent the 95% confidence interval.

In order to identify potential time-varying changes in monolingual and bilingual infants' attention to ID vs. AD faces throughout the duration of the preferential viewing paradigm, a series of *t*-tests were conducted for each 1000 ms epoch for each trial. Data from each epoch were then averaged across participants and across trials to create a dataset with 20 bins. Two *t*-tests were conducted per bin. One compared monolingual vs. bilingual looking to the ID face; the second compared monolingual vs. bilingual looking to the AD face. The 95% confidence interval was calculated for the data and a False Discovery Rate (FDR; Benjamini, 2010) analysis was subsequently carried out to correct for multiple comparisons. As assessed by pairwise *t*-tests and FDR corrections, there was no time point within the clip where monolinguals and bilinguals differed from each other in their attention to either ID or AD faces (see Figure 2).

## Experiment 1B

Experiment 1A revealed that monolingual and bilingual 3-month-olds showed similar patterns of looking to ID and AD faces in varying speech conditions. To investigate the potential influence of language during Experiment 1A, in Experiment 1B we analyzed data from Kim and Johnson (2013), which used the same ID and AD paired-face paradigm but had no sound in any trial. The goal of this study was to examine whether monolingual and bilingual infants engage in different looking patterns when presented with infant-directed (ID) and adult-directed (AD) faces side-by-side in silence. Again, we predicted that bilingual infants, more than monolingual infants, would tend to divide their attention between both faces. As in Experiment 1A, we measured infants' dwell time and number of fixations to each face.

### Method

#### Participants

Participants were classified as monolingual or bilingual using the same procedure as explained in Experiment 1A. Sixteen monolingual (8 girls) and 11 bilingual (8 girls) 6-month-olds (*M* = 5.91 months, *SD* = 0.37 months) participated in this study. Monolingual infants had an average of 0.5% L2 exposure (range: 0–1%), whereas bilingual infants had an average of 25% L2 exposure (range: 10–40%). Seventeen infants were excluded from the original data set due to a lack of language experience information.

#### Design

This experiment used a 2 × 2 mixed design. Language group (monolingual or bilingual) served as the between-subjects factor, and face type (ID or AD) was the within-subjects factor. Dependent variables of interest were dwell time (DT) and number of fixations to each face.

#### Stimuli and apparatus

Eye movement data were collected using a Tobii T60 XL eye-tracker. Eye movements were recorded at approximately 60 Hz with 0.5–1° spatial accuracy. Infants were seated on a parent's lap approximately 60 cm from the 24″ monitor on which stimuli were presented. The calibration procedure was the same as detailed in Experiment 1A.

**Figure 3:**
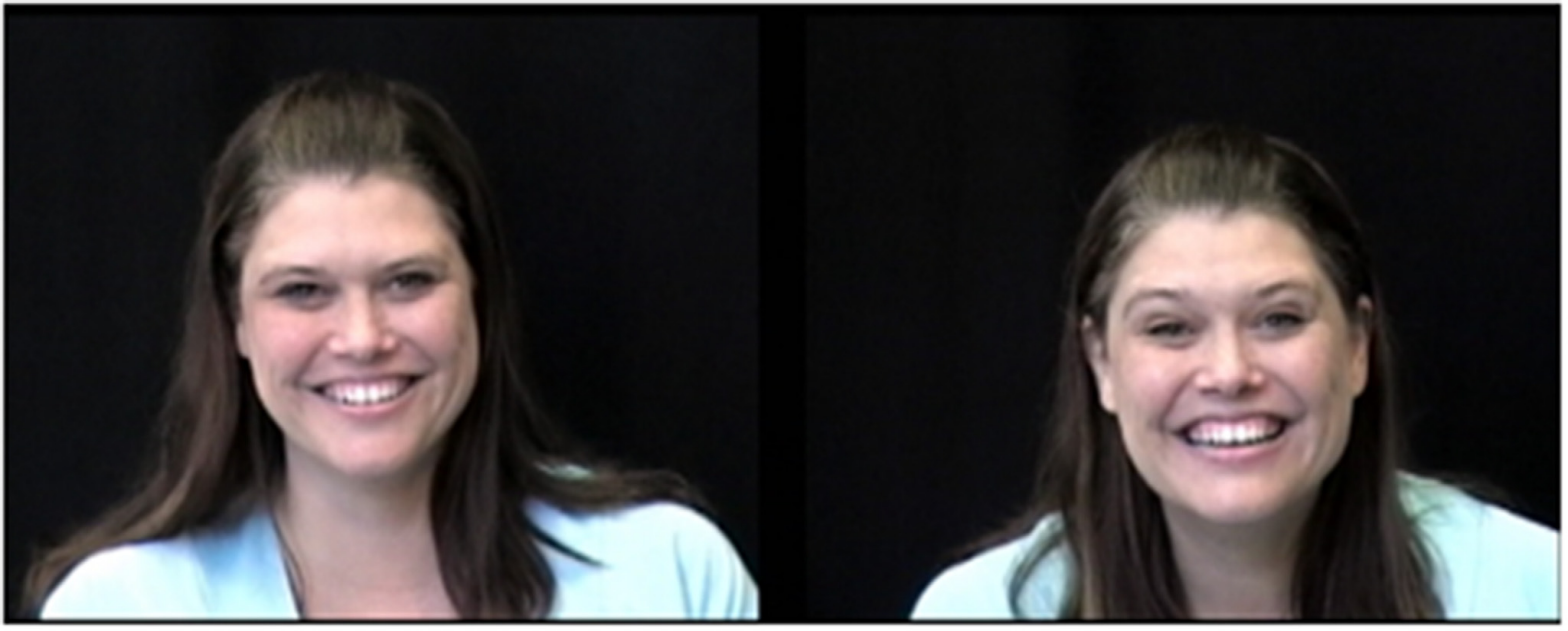
**Screen shot of the video stimuli used in Experiment 1B**. As in Experiment 1A, infants viewed both videos simultaneously on the same screen; in contrast to Experiment 1A, all videos were viewed in silence. The ID face is on the right, and the AD face is on the left.

To create the stimuli, experimenters recorded videos of the same woman describing a happy event to either an adult (her husband) or an infant (her 18-month-old). Each video was divided into several 10-s segments, which were then rated as being infant-directed or adult-directed by undergraduates. Six ID and six AD segments were then paired to create six unique video pairs (see Figure 3). Areas of interest (AOIs) were drawn generously in order to accommodate both face movement and possible eye tracker inaccuracies. Detailed information about stimuli creation can be found in Kim and Johnson (2013).

#### Procedure

Infants were seated on a parent's lap for the duration of the study. In each 10-s trial, two side-by-side silent videos were shown. Before each new trial, a visual attention-getter appeared in the center of the monitor to re-orient the infant's gaze to the middle of the screen. Each of the six video pairs was shown twice, creating 12 trials in all.

### Results and discussion

In this experiment, our main research question was whether 6-month-old infants' language experience influenced their looking to ID and AD faces. If infants in either language group showed a preference for ID over AD faces, this would extend the well-documented findings of infants' preference for ID speech (e.g., Cooper and Aslin, 1990) to include ID stimuli from visual modalities as well. Infants' looking times to each face were converted into proportions based on the total trial length (i.e., their total possible looking time); to control for individual differences in looking time, total dwell time was used as a covariate. However, because this variable is a time-varying covariate, the 2 × 2 ANCOVA appropriate to this design could not be carried out. Instead, we used a linear regression as the appropriate statistical procedure to examine language experience on attention to ID vs. AD faces. The model was defined by the Linear Regression procedure in SPSS with language experience and face type as the predictor variables used and proportion of dwell time as the criterion variable. This model explains 10.9% of the variance of proportion of dwell time and is marginally significant [*F*_(2, 53)_ = 3.10, *p* =.053; see Table 3]. There was not an effect of language experience, over and above the effect of face type [*B* = 0.01, *t*_(51)_ = 0.40, *p* = 0.691]. However, there was a significant effect of face type in the model [*B* = −0.63, *t*_(51)_ = −2.46, *p* = 0.017] with all infants exhibiting a greater proportion of dwell time directed at ID than AD faces.

**Table 3:** **Mean proportion dwell time to ID and AD faces in Experiment 1B**.

	**ID face**	**AD face**
Monolingual	0.307 (0.08)	0.374 (0.09)
Bilingual	0.323 (0.07)	0.379 (0.14)

*Standard deviations appear in parentheses*.

**Figure 4:**
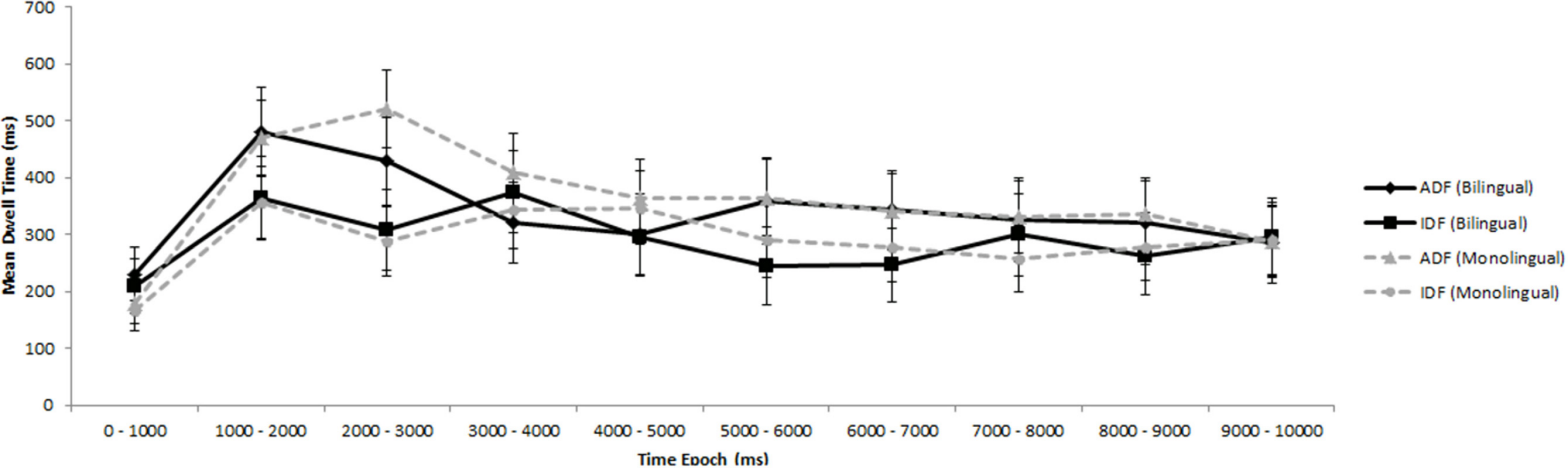
**Infants' looking patterns in Experiment 1B**. The solid black line represents bilingual infants' looking patterns to the adult-directed face (ADF) and the infant-directed face (IDF); the dotted gray line represents monolingual infants' looking patterns. Error bars represent the 95% confidence interval.

A similar set of time-varying analyses was conducted as in Experiment 1A to evaluate the possibility that attention to the face types changed by language background throughout the stimuli (see Results and Discussion for capitalize - 1A). In this case, because each trial was 10 s in duration, ten 1000 ms bins were created. Again, monolinguals and bilinguals did not differ from each other at any given epoch on their attention to ID and AD faces (see Figure 4). These results are consistent with Experiment 1A and further show that monolingual and bilingual infants do not differ in their attention to ID and AD faces.

## Experiment 2

Experiments 1A and 1B examined monolingual and bilingual infants' looking behavior when presented with social stimuli (two side-by-side faces) and found no consistent differences between the two groups. Experiment 2 expands the scope of the stimuli to include both social and non-social items. Our research question in Experiment 2 was whether monolingual and bilingual 6-month-old infants would show different preferences or looking behavior when presented with a six-item array of objects including items such as faces, natural objects (e.g., plants), and artifacts (e.g., tools; Gluckman and Johnson, 2013).

### Method

#### Participants

Participants were classified as monolingual or bilingual using the same procedure as described in Experiment 1A. The sample analyzed for this experiment consisted of 5 monolingual (2 girls) and 8 bilingual (2 girls) 6-month-old (*M* = 5.88 months, *SD* = 0.33 months) infants. Bilingual infants had an average of 35% L2 exposure (range: 20–50%); L2 exposure data were not available for monolingual infants. Seven infants were excluded due to a lack of language exposure information, and 12 infants were excluded due to technical error.

#### Design

This experiment used eye-tracking methods to examine 6-month-old infants' looking behavior when presented with a six-item array of objects. The between-subjects variable was language background (monolingual or bilingual). The dependent variable of interest was the total number of unique areas to which fixations were made.

#### Stimuli and apparatus

**Figure 5:**
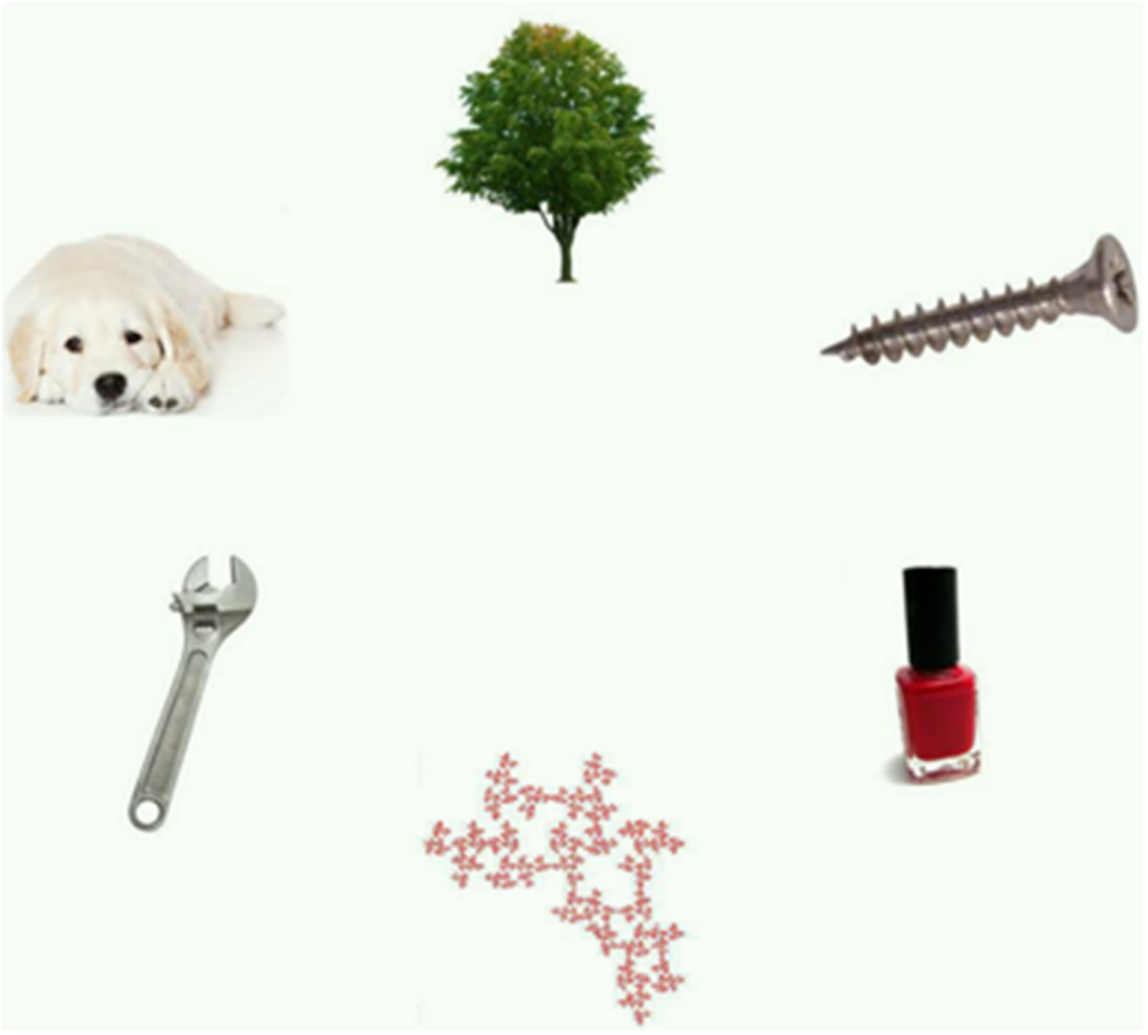
**Sample array of objects shown in Experiment 2**. Infants viewed a total of 48 arrays for 4 s each. Infants' fixations to each object were recorded.

A Tobii 1750 eye-tracker was used to collect eye movement data; see Experiment 1A for a detailed description of the setup and calibration process. In each trial, infants viewed a six-item array of objects from categories such as animals, body parts, faces, household objects, musical instruments, and mechanical objects (see Figure 5). Detailed descriptions of stimulus creation and presentation can be found in Gluckman and Johnson (2013).

#### Procedure

Infants were seated on a parent's lap for the duration of the experiment. Before each new trial, a visual attention-getter appeared in the center of the monitor to re-orient the infant's gaze to the middle of the screen. There were 48 trials, each with a duration of 4000 ms.

### Results and discussion

The main question of this study was whether monolingual and bilingual 6-month-old infants would show different preferences or looking behavior when presented with a six-item array of objects. To test the hypothesis that bilingual infants may engage in broader patterns of looking compared to monolinguals, we analyzed the number of unique object locations (areas of interest, or AOIs) to which fixations were made. Broader patterns of looking would be indicated by fixations to a greater number of unique AOIs. A One-way ANOVA revealed that monolinguals and bilinguals both looked at equal numbers of unique AOIs per trial (monolingual *M* = 2.92, bilingual *M* = 2.88), *F*_(1, 11)_ = 0.03, *p* = 0.858 (see Table 4). The results of this experiment add to our findings from Experiment 1 in that these results also show no difference in where monolingual and bilingual infants look when processing visual stimuli that contain both social and non-social information.

**Table 4:** **Mean number of unique AOIs viewed in Experiment 2**.

	**Number of unique AOIs viewed**
Monolingual	2.92 (0.31)
Bilingual	2.88 (0.34)

*Standard deviations appear in parentheses*.

## Experiment 3

The main research question in Experiment 3 was whether infants from monolingual and bilingual homes showed different patterns of visual attention when viewing complex scenes. In contrast to Experiments 1 and 2, Experiment 3 presented infants with stimuli in which there were no faces or social information. The stimuli consisted of photographs of scenes including objects such as boats, buildings, and trees. Although the results from Experiment 2 suggest that bilingual infants do not exhibit broader patterns of attention compared to monolingual infants, this could be due to the fact that the stimuli used in Experiment 2 are dissimilar to the natural, complex environments with which infants have the most visual experience. Thus, Experiment 3 further examines the question of broader attention by using photographs of natural, complex scenes.

### Method

#### Participants

Participants were classified as monolingual or bilingual as detailed in Experiment 1A. The final sample consisted of 19 monolingual (5 girls) and 22 bilingual (9 girls) infants, ranging in age from 3 to 15 months (*M* = 8.29 months, *SD* = 3.53 months). Monolingual infants had an average of 2.5% L2 exposure (range: 0–5%); bilingual infants had an average of 37.5% L2 exposure (range: 25–50%). Eighteen infants were excluded due to a lack of language exposure information, and 11 infants were excluded due to fussiness during the study.

#### Design

These data were collected as part of a larger study examining visual perception and visual search ability in infancy (Nguyen et al., 2012). In the part of the study relevant to Experiment 3, infants viewed a series of photographs of indoor and outdoor scenes which were specifically selected for their lack of social stimuli. Data for infants' dwell times and total number of fixations were collected.

#### Stimuli and apparatus

Eye movement data were collected with an SR EyeLink 1000 eye tracker. Eye movements were recorded at 500 Hz with an approximate spatial accuracy of 0.5–1°. Each infant sat on a parent's lap approximately 60 cm from a 22” ViewSonic VX2268wm monitor. To calibrate each infant's point of gaze, a dynamic attention-getter was briefly shown at five points around the screen in a crosshair pattern (top middle, bottom middle, left, right, and center). The calibration process was performed twice to ensure validity of the calibration.

Infants viewed a series of 28 photographs containing natural objects, such as trees and landscapes, or manmade objects, such as boats and buildings. Each photograph had a resolution of approximately 1024 × 768 pixels and dimensions of 27.2 × 20.25 cm.

#### Procedure

**Figure 6:**
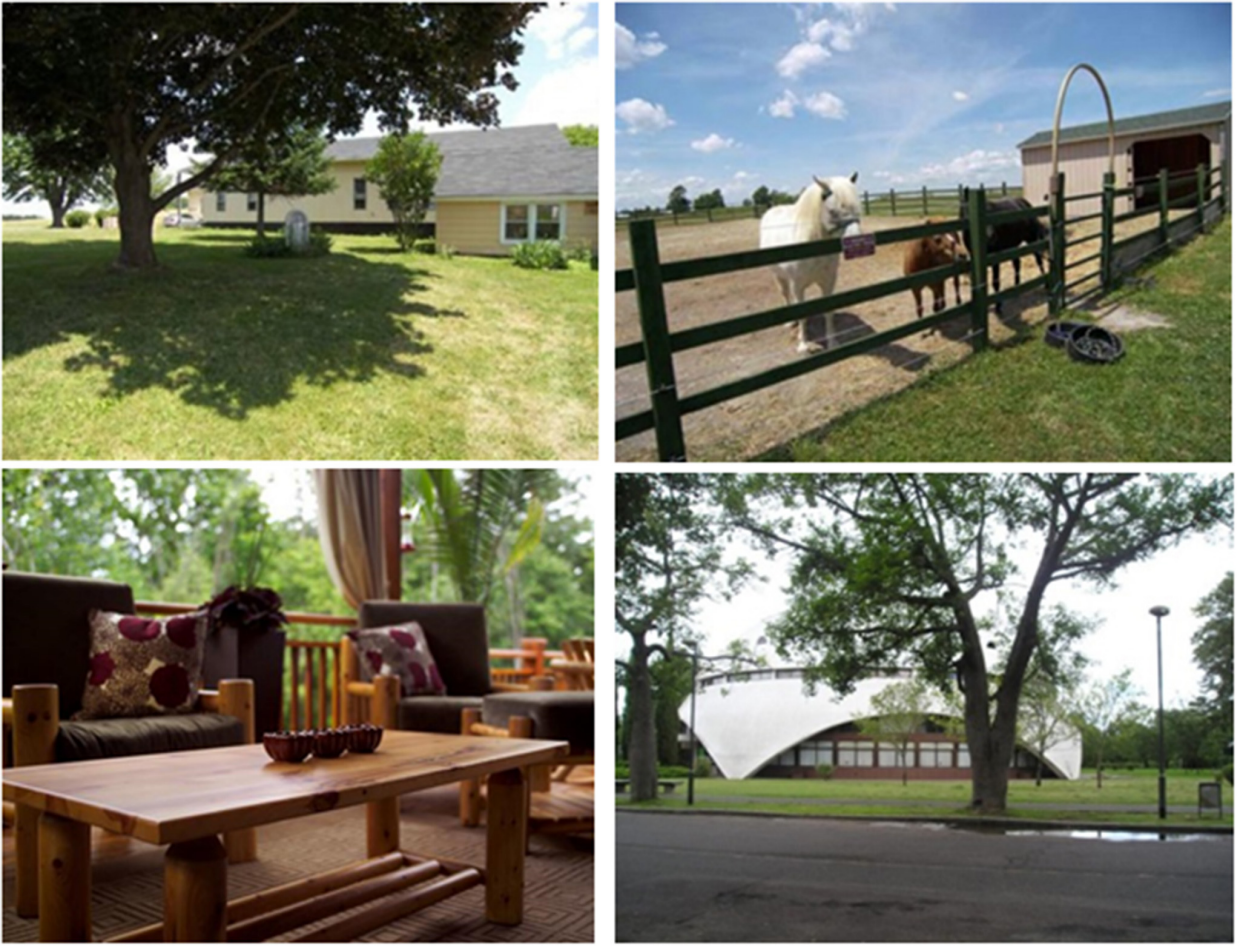
**Four of the 28 photographs used as stimuli in Experiment 3**. Infants viewed each photograph for 4 s and were allowed to look freely at each photograph during this time.

Infants were seated on a parent's lap for the duration of the experiment. In this portion of the experiment, infants viewed a series of 28 photographs. Before each new trial, a visual attention-getter appeared in the center of the monitor to re-orient the infant's gaze to the middle of the screen. In each trial, a static picture of a natural scene appeared for 4000 ms. See Figure 6 for a subset of the photographs that were used.

### Results and discussion

In this experiment, we analyzed infants' looking behavior for the photographs that they saw. Analyses of infants' looking behavior to a subsample of individual photographs showed that monolinguals and bilinguals did not differ in measures of dwell times in AOIs incorporating distinct objects, all *p*s > 0.05 (Table 5), or in the number of fixations made to any photograph, all *p*s > 0.05 (Table 5). Thus, bilingual and monolingual infants appear to have similar looking behaviors to non-social as well as to social stimuli.

**Table 5:** **Mean dwell time and number of fixations to photographs in Experiment 3**.

		**Dwell time**			**Fixations**		
		**Mean (ms)**	***F* value**	***p* value**	**Mean number**	***F* value**	***p* value**
Photo 1	Monolingual	3209.67 (862)	0.27	0.609	7.33 (3.01)	0.02	0.901
	Bilingual	2971.09 (916)			7.09 (4.09)		
Photo 2	Monolingual	3396.00 (306)	1.78	0.202	7.00 (2.76)	0.39	0.541
	Bilingual	2756.54 (1137)			7.82 (2.48)		
Photo 3	Monolingual	3774.00 (151)	1.38	0.258	7.67 (2.16)	1.12	0.307
	Bilingual	3440.91 (674)			8.82 (2.14)		
Photo 4	Monolingual	3174.00 (477)	1.62	0.222	6.83 (2.14)	1.17	0.297
	Bilingual	3492.36 (500)			9.36 (5.45)		
Photo 5	Monolingual	3396.00 (306)	1.78	0.202	6.33 (1.21)	0.004	0.950
	Bilingual	2756.55 (1137)			6.27 (2.15)		

*Standard deviations appear in parentheses. Degrees of freedom for all tests are (1, 15)*.

## Experiment 4

Experiments 1–3 examined monolingual and bilingual infants' dwell times and fixations to a variety of social and non-social stimuli and found no differences between the two language groups in terms of where the infants looked. However, measures of dwell time and fixations do not address all components of infants' looking behavior. In Experiment 4, we analyzed infants' saccadic eye movements across Experiments 1–3 in order to specify *how* infants processed the visual stimuli they saw. Although fixations and dwell time provide information about attention to specific areas, they do not provide information about patterns of looking. In Experiments 1–3, we found no evidence that bilinguals engage in broader looking when we analyzed fixations and dwell time; however, it is still possible that bilinguals' patterns of looking (i.e., saccades) differ from those of monolinguals. Specifically, based on our hypothesis that bilingual infants may pay broader attention to their environments, we predict that bilingual infants may have greater saccade amplitude (size) or frequency than monolingual infants.

### Method

#### Participants

Participants were classified as monolingual or bilingual using the same criteria as detailed in Experiment 1A. We analyzed saccades from all the infants who provided data for Experiments 1–3. We included any infants who provided saccade data, even if they ended their participation before the completion of the study (resulting in their exclusion from prior fixation and dwell time analyses). In total, there were 64 monolingual infants (31 girls; 24 boys; 9 with no gender data available) and 71 bilingual infants (33 girls; 29 boys; 9 with no gender data available) ranging in age from 3 to 15 months (*M* = 6.31 months, *SD* = 2.85 months). Monolingual infants had an average of 3.8% L2 exposure; bilingual infants had an average of 39.2% L2 exposure.

#### Identification of saccades

Identification of saccades from raw data began with identification of fixations, defined as accumulations of sampled gaze positions that remained within 0.5° visual angle for a minimum of 100 ms (Holmqvist et al., 2011). Eye-movement events that exceeded an instantaneous velocity of 30°/s for at least 33 ms between fixations (Baloh et al., 1975; Leigh and Zee, 1999) were flagged as saccades. Events for which the rate of change in pupil diameter was greater than two SD of the median rate of change for the entire sample, which would indicate a blink event, were excluded. Eye-movement events that met those criteria were considered saccades and the corresponding amplitude in degrees and maximum velocity were calculated.

### Results and discussion

The purpose of this experiment was to investigate whether monolingual and bilingual infants showed different patterns of looking behavior across a range of social and non-social stimuli. First, we analyzed whether language background played a role in the amplitude of infants' saccades. We hypothesized that if bilingual infants are scanning their environments more broadly, they may make larger or more frequent saccades. However, One-way ANOVAs showed no differences between monolingual and bilingual infants' saccade amplitudes (monolingual *M* = 7.44 degrees, *SD* = 1.85 degrees; bilingual *M* = 7.42 degrees, *SD* = 2.23 degrees), *F*_(1, 133)_ = 0.01, *p* = 0.942 (Table 6), or their saccade frequencies (monolingual *M* = 1.46 saccades per second, *SD* = 1.27 saccades per second; bilingual *M* = 1.38 saccades per second, *SD* = 0.48 saccades per second), *F*_(1, 133)_ = 0.290, *p* = 0.591 (Table 6). Experiment 4 adds to our conclusions from Experiments 1–3 by showing that in addition to there being no differences in fixations and dwell time between monolingual and bilingual infants, there are also no differences in patterns of looking.

**Table 6:** **Monolingual and bilingual infants' saccade characteristics in Experiment 4**.

	**Mean saccade amplitude (degrees)**	**Mean saccade frequency (saccades per second)**
Monolingual	7.44 (1.85)	1.46 (1.27)
Bilingual	7.42 (2.23)	1.38 (0.48)

*Standard deviations appear in parentheses*.

### General discussion

The experiments in this paper investigated the possibility that infants from monolingual and bilingual backgrounds attend to different kinds of visual features in social (Experiment 1), mixed social and non-social (Experiment 2), and non-social (Experiment 3) stimuli and whether these groups of infants show different patterns of looking overall (Experiment 4). Through our analysis of fixations, dwell times, saccade amplitude, and saccade frequency, we found no reliable differences between monolingual and bilingual infants' looking behaviors at any age. This series of experiments adds to the existing literature on bilingual input during infancy by showing that, in contrast to speech perception (e.g., Fennell et al., 2007), language background does not appear to affect looking behavior to visual stimuli. Our results are especially interesting given that language experience plays a role in visual attention and perception later in life (e.g., Davidoff, 2001; Dils and Boroditsky, 2010; Yow and Markman, 2011).

One explanation for the similarities found between language groups is that infants at the ages tested have not yet had enough language experience for any differences to appear in the ways that they attend to their environments. Each experiment in this paper included preverbal infants between the ages of 3 and 15 months. As infants become older, their attentional proclivities may change as they begin attending more to language and as they develop receptive and productive vocabularies (cf. Lewkowicz and Hansen-Tift, 2012). Thus, differences between infants from monolingual and bilingual backgrounds may emerge later in infancy than the ages we tested. Future research should investigate the possibility of perceptual differences in 15–20-month-old infants, as infants in this age range are likely to have begun producing words but are still not fluent in any language.

Alternatively, it may be that a productive vocabulary is necessary before differences between monolinguals and bilinguals (e.g., in cognitive tasks; Bialystok and Martin, 2004; Carlson and Meltzoff, 2008; Brito and Barr, 2012) begin to develop. However, it is unknown how cognitive differences relate to size or fluency of productive vocabulary. Future research should investigate this potential relation.

We must also consider the possibility that there really are no meaningful differences in the ways that monolingual and bilingual infants process visual information. If this is the case, it would suggest that the cognitive differences found later in life are not related to visual perception in infancy. Future research examining older infants' behaviors as well as the influence of productive vocabulary will help to create a more complete picture of bilingual cognitive development and help to pinpoint the developmental time point at which these cognitive differences begin to emerge.

The findings presented in this paper add to our knowledge about the role of language experience in early perceptual development. Some studies of infants and children have found that the experience of being bilingual leads to cognitive advantages (see Bialystok and Martin, 2004; Bialystok and Feng, 2009; Kovács and Mehler, 2009); however, these advantages may be limited to particular tasks, contexts, or age groups, as a bilingual advantage is not always found (see Hilchey and Klein, 2011; Paap and Greenberg, 2013; Gathercole et al., 2014). In order to resolve this ongoing debate, a more thorough analysis of the perceptual precursors to these potential cognitive advantages is needed. Future research examining the influence of language background on basic perception and learning early in development will be instrumental in helping to resolve this debate and in creating a more complete picture of early bilingual development.

### Conflict of interest statement

The authors declare that the research was conducted in the absence of any commercial or financial relationships that could be construed as a potential conflict of interest.
