# Supplementary material for: Does bilingual experience affect early visual perceptual development?
Source: Front Psychol. 2014 Dec 11;5:1429. doi: 10.3389/fpsyg.2014.01429 (PMC4263081; doi:10.3389/fpsyg.2014.01429)
Supplement: Supplementary file 1 [file PrimaryCaregiverQuestionnaire.DOCX]

Appendix A: Primary Caregiver Questionnaire

**PRIMARY CAREGIVER QUESTIONNAIRE**

1. Please list the people who lived at home (e.g., mother, father, grandmother, cousin) and/or cared for your child when your child was 0-AGE AT STUDY months old and the age your child was during this time.

| **Relationship to child** | **Child’s age (months) during residence** |
| --- | --- |
| *ex. Grandmother* | *2-5* |
|  |  |
|  |  |
|  |  |

1. If your child has any siblings, please list their birthdates: ________________________ ______________________________________________________________________
2. Think back to when your child was 0- AGE AT STUDY months old. What languages were spoken to your child, and how often?

| **Language** | **Percentage of the time** |
| --- | --- |
| *ex. German* | *50%* |
|  |  |
|  |  |
|  |  |
|  |  |

1. Please list the languages spoken by each person in your home when your child was 0- AGE AT STUDY months old, and mark the degree of fluency for each.

| **Person** | **Language** | **Fluency (1 = low, 5 = high)** |
| --- | --- | --- |
|  |  | 1 2 3 4 5 |
|  |  | 1 2 3 4 5 |
|  |  | 1 2 3 4 5 |
|  |  | 1 2 3 4 5 |
|  |  | 1 2 3 4 5 |
|  |  | 1 2 3 4 5 |
